# Fluorescent Image Analysis of HIV-1 and HIV-2 Uncoating Kinetics in the Presence of Old World Monkey TRIM5α

**DOI:** 10.1371/journal.pone.0121199

**Published:** 2015-03-24

**Authors:** Eri Takeda, Ken Kono, Amy E. Hulme, Thomas J. Hope, Emi E. Nakayama, Tatsuo Shioda

**Affiliations:** 1 Department of Viral Infections, Research Institute for Microbial Diseases, Osaka University, Suita, Osaka, Japan; 2 Department of Cell and Molecular Biology, Feinberg School of Medicine, Northwestern University, Chicago, Illinois, United States of America; University of Colorado Denver, UNITED STATES

## Abstract

Uncoating of Human Immunodeficiency Virus type 1 (HIV-1) and type 2 (HIV-2) conical cores is an important early step for establishment of infection. In Old World Monkey (OWM) cells, the TRIM5α cellular factor potently suppresses an early step of infection by HIV-1. Previously, biochemical studies using whole cell lysates of infected cells revealed that OWM TRIM5α accelerates the uncoating of HIV-1, leading to premature reverse transcription. In the present study, we re-evaluated uncoating kinetics of HIV-1 in the presence of OWM TRIM5α by using an *in situ* uncoating assay, which allowed us to differentiate productive HIV-1 entry from simple (non-productive) endocytosis. Results showed that the uncoating kinetics of HIV-1 was indeed accelerated in the presence of OWM TRIM5α. Furthermore, we adapted an *in situ* uncoating assay to HIV-2, which showed wide variations in TRIM5α sensitivity among different isolates. HIV-2 isolate GH123, whose infectivity was suppressed by cynomolgus monkey (CM) TRIM5α, showed accelerated uncoating in the presence of CM TRIM5α. In contrast, mutant HIV-2 ASA, whose infectivity was unaltered by CM TRIM5α, showed no change in uncoating kinetics in the presence of CM TRIM5α. These results confirmed and further extended the previous notion that accelerated uncoating is associated with restriction activity of TRIM5α against lentiviruses.

## Background

Uncoating of the lentivirus core, which is composed of ∼1,000 capsid proteins (CA), is an important process for establishment of viral infection. Human Immunodeficiency Virus (HIV) infection begins with the binding of viral glycoprotein to the cellular receptor and co-receptors, a step that is followed by fusion of the viral and cellular membranes. After the fusion, a conical core that contains two viral genomic RNAs and several viral proteins is released into the cytoplasm of the target cell. In the cytoplasm, CAs eventually dissociate from the viral complex in a process termed uncoating. During the uncoating process, reverse transcription (RT) of the viral genomes is initiated. The resulting double-stranded DNA is associated with viral and cellular proteins, forming a structure designated the pre-integration complex (PIC). The PIC migrates into the nucleus, where viral DNA integrates into the chromosomal DNA of the target cell. Several studies have reported that mutations in the HIV type 1 (HIV-1) CA-encoding gene affect viral core stability [1–4]. Changes in core stability caused by some of these CA mutations seem to affect uncoating kinetics, which may result in impaired RT or nuclear entry. Thus, timely uncoating is thought to be important for efficient HIV-1 infection.

To analyze uncoating kinetics of HIV-1 in infected cells, Campbell *et al*. developed an *in situ* uncoating assay [5] by using fluorescently labeled HIV-1. In that assay, HIV-1 was double-labeled using a green fluorescent protein (GFP) fused with viral protein Vpr (GFP-Vpr) along with a protein consisting of the amino-terminal 15 amino acids of the Src protein (S15) fused with a red fluorescent protein (RFP). S15 contains a signal peptide for membrane trafficking of Src, and therefore directs the fused RFP to the plasma membrane and viral envelope. The RFP signals in HIV-1 were observed to disappear after productive entry of the virus into the host cell. The infected cells then were fixed and stained with a Cy5-labeled antibody detecting HIV-1 p24 CA; the fluorescent signal was analyzed using fluorescence microscopy. The total complexes that entered the cytoplasm (green spots that lost red signals) were counted, and the number of complexes that contained CA (coated) was compared to the number of complexes that lost CA staining (uncoated). This methodology revealed a relationship between replicative capability and uncoating kinetics of HIV-1 CA mutant viruses [2,4] along with a relationship between reverse transcription and uncoating of HIV-1 [6].

HIV-1 infects humans but not Old World Monkeys (OWM) such as Rhesus monkey (Rh) and cynomolgus monkey (CM). One intracellular antiviral factor, TRIM5α (tripartite motif protein 5α), was identified by the screening of an Rh-cDNA library [7]. Members of the TRIM protein family share RING, B-box, and coiled-coil domains; the alpha isoform of TRIM5 additionally includes a C-terminal PRYSPRY (B30.2) domain [8,9]. Though the *in vivo* activity of TRIM5α has not been investigated in detail, this isoform has been shown to recognize the CA conical core of invading viruses via the species-specific B30.2 domain [10–12]. Restriction ability of TRIM5α homologs varies among species of OWMs. Rh and CM TRIM5α homologs restrict HIV-1 but not Simian Immunodeficiency Virus isolated from macaque (SIVmac) [7,10], whereas African green monkey (AGM) TRIM5α restricts both HIV-1 and SIVmac [10,13]. In contrast, human (Hu) TRIM5α only weakly restricts HIV-1, but the Hu homolog potently restricts N-tropic murine leukemia virus (N-MLV) [13,14]. Interestingly, mutations in CA differentially affect viral sensitivity to TRIM5α from several species. Rh and Hu TRIM5α homologs reportedly facilitate the destruction of the HIV-1 and N-MLV cores, respectively [15,16].

In the case of HIV type 2 (HIV-2), we previously reported that amino acid residue 120 of the CA encoded by the GH123 strain of HIV-2 determines viral sensitivity to CM TRIM5α [17,18]. The original GH123 strain encodes a proline residue at the 120^th^ position of CA, and the replication of GH123 is potently suppressed in the presence of CM TRIM5α. In contrast, mutant GH123 viruses encoding an alanine or glutamine at the 120^th^ position can grow efficiently in the presence of CM TRIM5α [17]. (Note that amino acid 120 in GH123 corresponds to position 119 in other strains of HIV-2.) Furthermore, a community-based cohort study (performed in Caio, Guinea-Bissau, in west Africa) revealed that CA contains 3 proline residues, at positions 119, 159, and 178 (residues 120, 160, and 179 in strain GH123), that were significantly associated with reduced loads of plasma HIV-2 [19]. The proline residues at these three positions also conferred slightly increased sensitivity to Hu TRIM5α [19].

In the present study, we adapted the *in situ* uncoating assay to HIV-2, and compared uncoating rates of HIV-1 strain NL4-3, HIV-2 strain GH123, and mutant HIV-2 carrying non-proline residues at positions 120, 160, and 179 in the presence and absence of AGM, Rh, CM, and Hu TRIM5α homologs.

## Materials and Methods

### Vector construction

Fragments encoding CM, Rh, AGM, and Hu TRIM5αs C-terminally tagged with hemagglutinin (HA) were amplified by PCR using the oligonucleotide primers 5’-GGCAGCTGGCCACCATGGCTTCTGG-3’ and 5’- CCGCGGCCGCTCAAGCAGCATAATCAG-3’ from the cDNA clones described previously [10,20]. These DNA fragments were cloned separately into the *Pvu*II/*Not*I-sites of the pCEP4 vector (Invitrogen), which carries the hygromycin B resistance gene. GH123-Nh is a mutant of the GH123 proviral clone in which an *Nhe*I restriction enzyme cleavage site at position 6183 was blunted and re-ligated, introducing frame-shift mutations in the *env* gene. ASA-Nh is a mutant of GH123-Nh in which the CA-encoding gene has been mutated to encode a capsid protein with substitutions of alanine, serine, and alanine for the proline residues at positions 120, 160, and 179, respectively. The HIV-2 GH123 ASA CA-encoding fragment derived from ASA-Nh [19] was inserted into the *Pml*I/*Bsu*36I-sites of the green fluorescent protein (GFP)-encoding HIV-2 construct GH123-GFP, which was a pROD-env(-)-GFP derivative carrying the gene encoding GH123 CA [18].

### Cell culture, DNA transfection, and establishment of stably transfected cell lines

Human embryonic kidney (HEK) 293T cells (ATCC CRL-11268) and HeLa cells (ATCC CCL-2) were maintained at 37°C in a 5% CO_2_ incubator in Dulbecco’s Modified Eagle Medium (DMEM; Nacalai Tesque, Kyoto, Japan) supplemented with 10% (v/v) heat-inactivated fetal bovine serum (FBS; Sanko Junyaku Co., Ltd., Tokyo, Japan), and penicillin/streptomycin (GIBCO, Auckland, NZ). HeLa cells were transfected with pCEP4 encoding HA-tagged TRIM5α from each of the species or pCEP4 empty vector (Vc) by using the Lipofectamine LTX reagent (Invitrogen, Carlsbad, CA) according to the manufacturer’s recommendations. For the establishment of stably transfected cell lines, transfected cells were selected at 500 μg/ml hygromycin B (Invitrogen), and surviving cells were propagated in the presence of hygromycin B.

### Detection of HA-tagged TRIM5α in stably transfected cell lines

Expression of HA-tagged TRIM5α protein in the stably transfected cell lines was confirmed by immunoblot analyses of the cell lysates and by immunofluorescence staining of the cells. For immunoblotting, cells were lysed in sodium lauryl sulfate (SDS) sample buffer (100 mM sodium phosphate, pH 7.2, 1% SDS, 10% glycerol, 100 mM dithiothreitol, and 0.001% bromophenol blue). The lysates were heat-denatured and loaded onto a 4–12% gradient SDS-polyacrylamide gel (Invitrogen), and separated proteins were electronically transferred to a polyvinylidene difluoride membrane. Following blocking (1 hr at room temperature in phosphate-buffered serine (PBS) + 0.05% (v/v) Tween 20 (PBST) containing 5% skim milk), membranes were incubated (2 hr at room temperature) with rat anti-HA monoclonal antibody (Roche, Indianapolis, IN) or rabbit anti-actin polyclonal antibody (Sigma-Aldrich, St. Louis, MO) diluted in PBST containing 3% skim milk. The membranes were washed with PBST and incubated with horse radish peroxidase (HRP)-conjugated goat anti-rat IgG (for anti-HA; American Qualex, San Clemente, CA) or protein G (for anti-actin; Sigma-Aldrich) diluted in PBST containing 3% skim milk. After washing with PBST, bound antibodies were visualized with the ChemiLumi-One L chemiluminescent kit (Nacalai Tesque, Kyoto, Japan). Gel images were captured with Ez-Capture (ATTO, Tokyo, Japan). For immunofluorescence staining, 2 × 10^5^ HeLa cells harboring CM TRIM5α-encoding plasmid (or Vc) were fixed with 3.7% formaldehyde/PBS for 15 min and then incubated with 0.2% Triton X-100 for 7 min. HA-tagged TRIM5α was detected by incubation with anti-HA antibody diluted in PBS containing 3% skim milk for 2 hr. Cells then were rinsed with PBS, and incubated with FITC-conjugated goat anti-rat IgG (for anti-HA, American Qualex, San Clemente, CA) diluted in PBS containing 3% skim milk. After staining of nuclei with Hoechst 33342 (Dojindo Laboratories, Kumamoto, Japan), cells were observed by fluorescence microscopy using an Eclipse TE2000-E microscope (Nikon Corporation, Tokyo, Japan).

### Virus propagation

Infectious viruses encoding GFP were prepared by transfection (using polyethyleneimine; PEI) of HEK293T cells with proviral DNA clones (HIV-1-based MsMnG [21], HIV-2-based GH123-GFP, or ASA-GFP) together with the pMD2G plasmid encoding vesicular stomatitis virus envelope protein (VSV-G). VSV-G-pseudotyped HIV-1 and HIV-2 viruses for quantitative PCR (qPCR) analyses were prepared by PEI-mediated co-transfection of HEK293T cells with *env*-deficient NL-Nh, GH123-Nh, or ASA-Nh together with pMD2G. The resulting viruses were treated with DNase I before use in HeLa cell infection. Production of fluorescence-labeled viruses for the *in situ* uncoating assay was described previously [2,4,5]. Briefly, labeled HIV-2 was generated by co-transfection into HEK293T cells with *env*-deficient GH123-Nh or ASA-Nh, S15-dTomato, VSV-G, and GFP-Vpx (a fusion protein of GFP and Vpx derived from SIV PBj14 [GenBank accession number: M31325]). Viral titers were determined by measuring the concentration of HIV CA protein using the RETROtek antigen ELISA kit (ZeptoMetrix, Buffalo, NY).

### Viral infection assay

HeLa cells (5 x 10^5^) transfected with HA-tagged TRIM5α-encoding vector (or with Vc alone) were infected with viral titers of the HIV-GFP derivative equivalent to 40 ng CA. After 24 hr infection, cells were trypsinized and fixed with 3.7% formaldehyde/PBS. After changing the buffer to PBS, GFP-expressing cells were counted by flow cytometry (Becton, Dickinson and Company, Franklin Lakes, NJ).

### Quantitation of HIV DNA genomes in TRIM5α-expressing cells

HeLa cells (2 × 10^5^/well) transfected with vector encoding TRIM5α from each of several species (or with Vc alone) were seeded in 24-well plates on the day before the experiment. Cells were spinoculated with viral titers of VSV-G-pseudotyped HIV-1 and HIV-2 equivalent to 200 ng CA for 2 hr at 16°C. Cells were washed with PBS and incubated in fresh medium for 1 or 3 hr at 37°C in a 5% CO_2_ incubator. After treatment of cells with RNase A, DNA was extracted from infected cells using a GenElute Mammalian Genomic DNA Miniprep Kit (Sigma-Aldrich). Approximately 100 ng of purified DNA was used in qPCR with the KOD SYBR qPCR Mix (TOYOBO) according to the manufacturer's protocol using a 7500 Fast Real-Time PCR System (Applied Biosystems, Foster City, CA). PCR conditions were as follows. After initial incubations at 98°C for 2 min, 40 cycles of amplification were carried out for 10 sec at 98°C and 10 sec at 60°C (primer set: HIV-1 *GAG*, HIV-2 *GAG* and human *GAPDH*), 56°C (HIV-1 2-LTR) or 62°C (HIV-2 2-LTR) followed by 1 min at 68°C. Following amplification, the melting curves were determined on the SYBR channel. Primer sequences were as follows: HIV-1 *GAG* primers, 5’-GCAGCCATGCAAATGTTAAAAGAG-3’ (forward) and 5’-TCCCCTTGGTTCTCTCATCTGG-3’ (reverse) [22,23], HIV-1 2-LTR primers, 5’-GTGCCCGTCTGTTGTGTGACT-3’ (forward) and 5’-CTTGTCTTCTTTGGGAGTGAATTAGC-3’ (reverse) [22,23], HIV-2 *GAG* primers, 5’-AGGCCACAAAATCCCGTGCCG-3’ (forward) and 5’-ACGGGGAAGTTGCGGGGCTT-3’ (reverse), HIV-2 2-LTR primers, 5’-TGTTCCCTGCTAGACTCTCACCAGTGC-3’ (forward) and 5’-CCTGGCCCATGAGTATAATTCTGCCAATCTGG-3’ (reverse), and human *GAPDH* primers (for use as an internal control), 5’-GAGCTCAACGGGAAGCTCACT-3’ (forward) and 5’-GTCAAAGGTGGAGGAGTGGG-3’ (reverse). Genomic copy numbers were determined from threshold cycle values following qPCR by using the standard curve of each gene copy, and then expressed as copies per μg total DNA using the copy numbers of the internal control DNA, the human GAPDH-encoding gene, in the HeLa genomic sample. For the standard curve of HIV-2 2-LTR circles, the HIV-2 2-LTR circle junction was cloned into a TA cloning vector by PCR amplification from the 2-LTR circles, using total DNA extracted from HeLa cells infected with VSV-pseudotyped HIV-2 GH123-Nh as the template for the reaction. In this method, levels of qPCR signals were well correlated with DNA copy numbers between 10 and 10^7^ copies (*r* ^*2*^ = 0.985, HIV-1 *GAG*; *r* ^*2*^ = 0.992, HIV-1 2-LTR; *r* ^*2*^ = 0.995, HIV-2 *GAG*; and *r* ^*2*^ = 0.987, HIV-2 2-LTR).

### *In situ* uncoating assay

The *in situ* uncoating assay was conducted as previously described [2,4,5]. Briefly, 2.2 × 10^5^ HeLa cells were infected with VSV-G-pseudotyped and fluorescently labeled HIV-1 or HIV-2 at viral titers equivalent to 100 ng of CA (p24 and p26, respectively). Specifically, HIV particles contained S15-dTomato and GFP-Vpr (HIV-1) or GFP-Vpx (HIV-2). In the present study, we used dTomato as a RFP. HeLa cells in 24-well plates were spinoculated with the labeled virus for 2 hr at 16°C in the presence or absence of bafilomycin A (BafA) (Sigma-Aldrich). Virus-containing supernatant then was removed and replaced with prewarmed medium in the presence or absence of BafA, incubated at 37°C for the indicated time interval, and then fixed with 3.7% formaldehyde (Polysciences, Warrington, PA) in 0.1 M PIPES buffer (pH 6.8). The fixed HeLa cells were permeabilized with blocking solution (PBS, 10% normal donkey serum [Jackson ImmunoResearch Laboratories, West Grove, PA], 0.01% Triton X-100, 0.01% NaN3) for 5 min at room temperature; subjected to primary staining for viral CA with anti-p24 mAb AG3.0 for HIV-1 (NIH AIDS Research and Reference Reagent Program) or anti-HIV-2 p26 (ImmunoDX, LLC, Woburn, MA) in blocking solution without Triton X-100 for 1 hr at room temperature; and subjected to secondary staining with Cy5-labeled donkey anti-mouse antibodies (Jackson ImmuoResearch Laboratories) for 30 min at room temperature. Images were collected and deconvolved with an Eclipse TE2000-E inverted microscope and NIS-Elements AR software (Nikon). Following deconvolution, viral GFP punctae were semi-automatically identified by individual GFP-intensity areas in the image picture, and Cy5- and dTomato-positive or -negative status was determined by each signal in the extractive GFP-area by using the NIS-Elements program.

### Statistical analysis

Differences in uncoating kinetics and GFP-positive area sizes were evaluated with unpaired t tests and Mann–Whitney *U* test, respectively.

## Results

### Establishment of HeLa cell lines stably expressing HA-tagged CM TRIM5α

**Fig 1:**
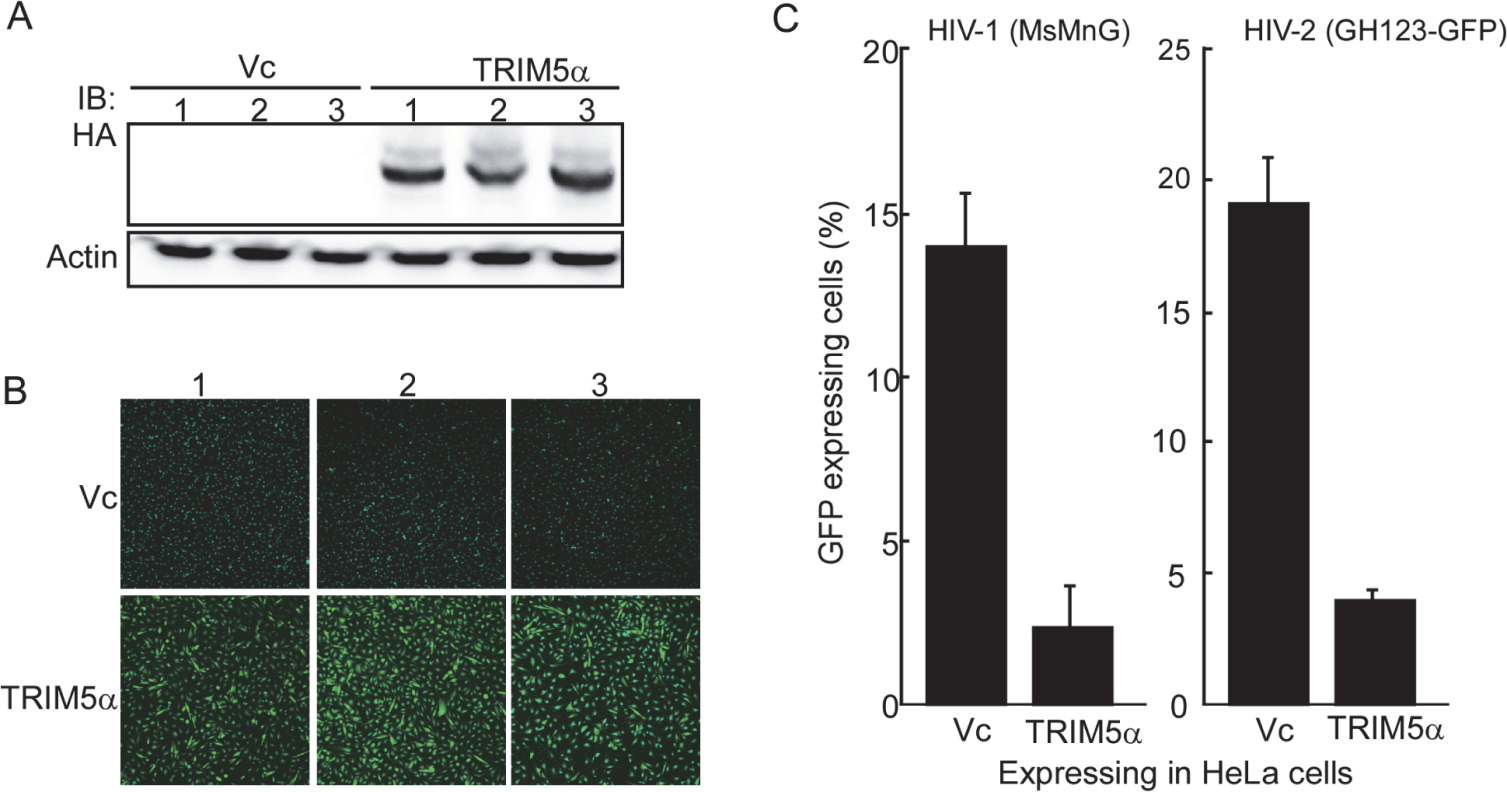
Establishment of HeLa cell lines stably expressing HA-tagged TRIM5α. HeLa cell lines were stably transfected with a vector control (Vc) or with a vector encoding HA-tagged cynomolgus monkey (CM) TRIM5α. (A) Cells from three independent cell lines were lysed and separated on a 4–12% gradient SDS-PAGE; intracellular HA-tagged proteins were visualized by immunoblotting using anti-HA antibody (upper panel) or anti-actin antibody (loading control; lower panel). (B) Three independent HeLa cell lines expressing HA-tagged CM TRIM5α were stained with anti-HA primary antibody and FITC-conjugated secondary antibody. Fluorescent signals (green) were observed by fluorescence microscopy (magnification power of 200). Nuclei (blue) were visualized by staining with Hoechst 33342. (C) HeLa cells transfected with Vc or with vector encoding HA-tagged CM TRIM5α were infected with GFP-encoding HIV-1 (MsMnG) or HIV-2 (GH123-GFP). At 24 hr after infection, cells were collected, fixed with 3.7% formaldehyde in phosphate-buffered saline, and sorted by flow cytometry (to detect GFP expression). Data are shown as means + SDs of three independent cell lines.

To analyze uncoating kinetics of HIV-1 and HIV-2 in the presence of OWM TRIM5α, we established HeLa cell lines stably expressing CM TRIM5α tagged with an HA epitope. HeLa cells were transfected with a HA-tagged CM TRIM5α-encoding construct based on the pCEP4 plasmid, a backbone vector that is known to provide constitutive protein expression in almost all mammalian cells. Transfected cells then were selected in culture medium containing hygromycin B. We established three independent cell lines stably expressing CM TRIM5α, along with control lines that harbored the empty vector (Vc). Fig. 1A shows results of immunoblot analysis of each cell line lysate using HA-specific antibody. HA-tagged CM TRIM5α proteins were readily detected at almost equal levels in the three HeLa cell lines transfected with the pCEP4-CM-TRIM5α-HA construct. Furthermore, the use of anti-HA antibody followed by FITC-labeled secondary antibody confirmed the presence of CM TRIM5α protein in virtually all transfectants (Fig. 1B, lower panels); green fluorescence was barely detectable in Vc transfectants (Fig. 1B, upper panel). In order to determine the restriction activity against HIV-1 and HIV-2 in these cell lines, we performed a single-round infection assay, in which viral infection was detected as fluorescence generated by production of the GFP encoded by the viral RNA (Fig. 1C). VSV-G-pseudotyped HIV-1 (MsMnG[21]) or HIV-2 (GH123-GFP [18]) viruses were inoculated into the HeLa cell lines expressing CM TRIM5α and into those transfected with Vc; at 24 hr after infection, cells were collected and fixed with formaldehyde. GFP-expressing cells were counted by flow cytometry; percentages of GFP-positive cells are shown in Fig. 1C. In the presence of CM TRIM5α, the numbers of GFP-positive cells were reduced to 17% (HIV-1) and 21% (HIV-2) of those in the control Vc cell lines. These results confirmed that HA-tagged CM TRIM5α retained anti-HIV activity when expressed in HeLa cells.

### Adaptation of *in situ* uncoating assay to HIV-2 and establishment of semi-automated fluorescent particle counting system

**Fig 2:**
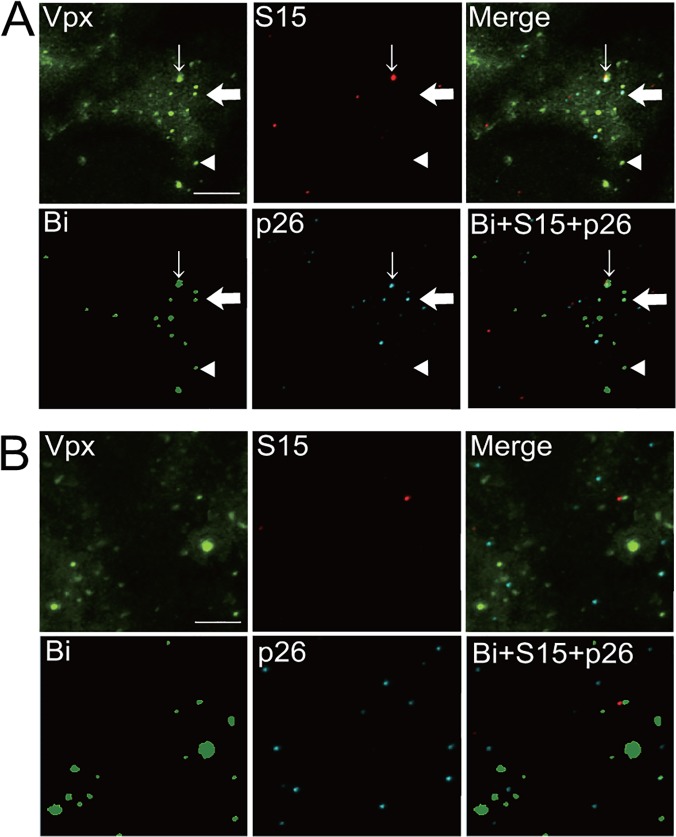
HIV-2 uncoating images. (A) Images of empty vector-transfected HeLa cells infected with fluorescently labeled HIV-2. The infected cells were fixed at 1 hr after infection. HIV-2 capsids (CA) in cells were probed with Cy5 using anti-HIV-2 p26 antibodies. Fluorescent images of GFP-Vpx (Vpx, green), S15-Tomato (S15, red), HIV-2 p26 (p26, blue), a merged image of these three colors (Merge), a binary image of GFP-Vpx (Bi, green), and a merged image of Bi, S15, and p26 (Bi+S15+p26) are shown. In this image, we detected one particle with red, green, and blue three-fluorescent co-localization (thin arrow); 4 particles with green and blue two-fluorescent co-localization (one of them is indicated by a thick arrow); and nine particles with green fluorescent only (one of them is indicated by an arrow head). A bar shows 10 μm. (B) Images of HeLa cells stably expressing cynomolgus monkey TRIM5α following infection with fluorescently labeled HIV-2. Layout of the images is as described in the legend for panel A. In this image, we detected no particles with red, green, and blue co-localized fluorescence; two particles with green and blue co-localized fluorescence; and 13 particles with green fluorescence only.

In the original *in situ* uncoating assay [2,4,5], HIV-1 was labeled with GFP-Vpr and S15-dTomato, and pseudotyped with VSV-G. Infection with labeled HIV-1 was synchronized by spinoculation. The S15-dTomato signal was expected to disappear after productive fusion of the virion with cellular endosomal membranes. At various times after infection, the cells were fixed and stained with an antibody against HIV-1 p24 CA. The cells then were analyzed by using fluorescence microscopy. The total complexes that entered the cytoplasm (green spots that lost S15-dTomato) were counted, and the number of complexes that contained CA (coated) was compared to the number of complexes that lost CA staining (uncoated). In order to adapt this *in situ* uncoating assay to HIV-2, we replaced GFP-Vpr with GFP-Vpx derived from SIV PBj14. We used replication-incompetent HIV-2 proviral clones carrying a frame-shift mutation in the *env* gene, since viruses had to be pseudotyped with VSV-G to permit entry into HeLa cells lacking the main HIV receptor CD4. Instead of the antibody against HIV-1 p24 CA, we used anti-HIV-2 p26 antibody to detect CA. Fig. 2A shows representative images of the *in situ* uncoating assay of HIV-2 in empty vector-transfected HeLa cells. The upper left, upper middle, and lower middle panels show fluorescence for GFP-Vpx (green), S15-dTomato (red), and HIV-2 p26 CA (blue), respectively, and the upper right panel shows a merged image for all three colors (Fig. 2A). Thin arrows indicate GFP-positive, S15-positive, CA-positive complexes (red, green, and blue) corresponding to intact virions. Thick arrows indicate GFP-positive, S15-negative, CA-positive complexes (green and blue) corresponding to virions that have productively entered cells but still remain coated. Arrowheads indicate GFP-positive, S15-negative, CA-negative complexes (green) corresponding to uncoated virions. In this representative image, we detected one intact virion, four virions that had productively entered cells but remained coated, and nine uncoated virions (Fig. 2A). S1 Fig. shows merged images with lower magnification, including the images shown in Fig. 2.

To facilitate counting of viral particles, and also to ensure more objective particle counting, we established a semi-automatic counting system in the *in situ* uncoating assay by using the NIS-element program. This system allowed us to count all GFP-positive particles above a certain threshold. In this system, GFP-positive viral particle images were converted to binary images and areas with fluorescent intensities above the background were determined for each experiment. Areas with positive signals sized between 0.07 and 2.0 μm^2^ then were selected automatically in the image (Fig. 2A, lower left panel). Locations of these areas were pasted on both original dTomato- and Cy5-visualized images. A merged image of the GFP binary image with S15- and CA- images is shown in the lower right panel of Fig. 2A. Fluorescent intensities per area were calculated automatically by the NIS-system, and the fluorescence-positive or -negative status was determined depending on the particle’s florescent intensity. In our previous report, we counted the number of particles in a subsection of the area of the images we obtained. This process was quite laborious, and there was no guarantee that the particles that were counted shared the same properties in all images. In this new semi-automatic counting system, however, we were able to take all GFP-positive particles in the obtained images into consideration. To confirm the equivalence of the new semi-automatic counting system and the previous manual method, we compared the results obtained by both methods (Table A in S1 File). This time, we manually counted all GFP-positive particles in the images. The correlation coefficients (*r* ^*2*^) were always over 0.98 when comparing between the direct manual counting and the semi-automatic system in terms of total GFP-positive particles (GFP-positive, S15-positive, and CA-positive particles (red, green, and blue; RGB); GFP-positive, S15-negative, and CA-positive particles (GB); and GFP-positive, S15-negative, and CA-negative particles (G)), GFP-positive viral particles that had productively entered the cytoplasm (GB and G), and CA-positive particles that had productively entered the cytoplasm (GB). Percentages of coated virus particles in productively entered particles (GB/ (GB+G) (%)) also showed good correlation (*r* ^*2*^ = 0.987). It should be noted here, however, that semi-automatic counting always missed some particle counts (average: 17.8% reduction) compared to direct manual counting. This discrepancy seemed to be due to the stringent automatic counting parameters; this restriction was implemented to preclude inclusion of particles with low-level signals or of those with large areas, which most likely corresponded to pairs of overlapping particles.

### Uncoating kinetics of HIV-1 and HIV-2 in CM TRIM5α-expressing HeLa cells

**Fig 3:**
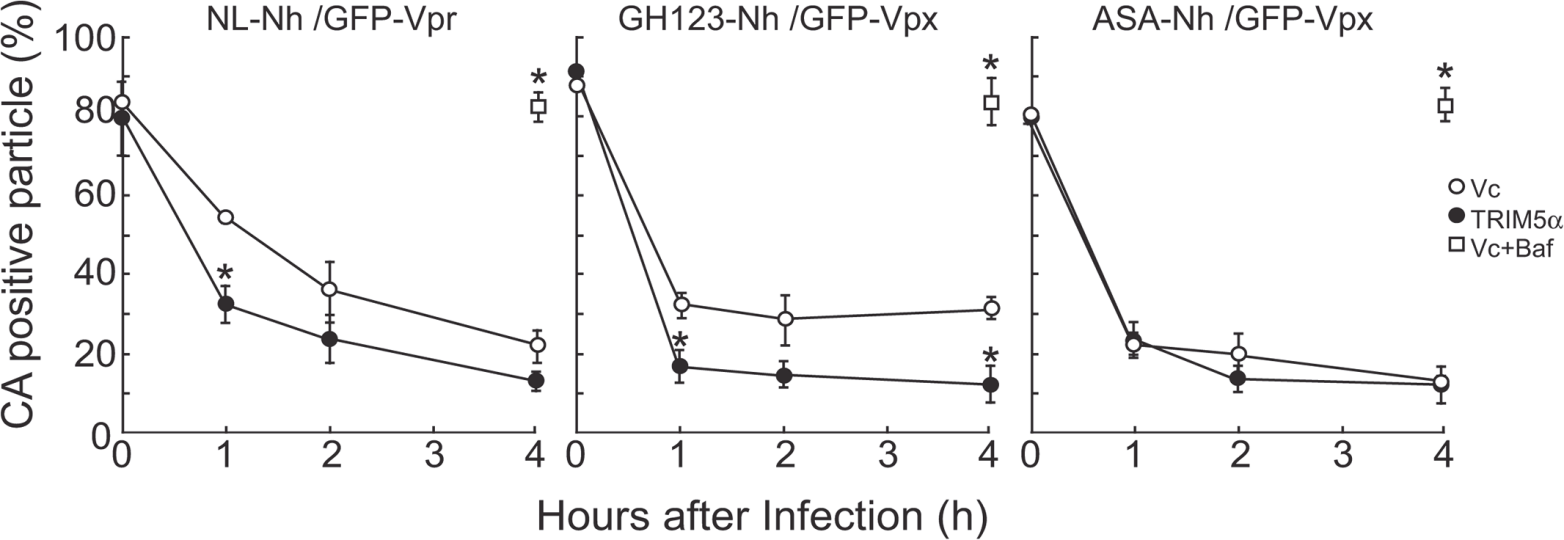
Uncoating kinetics of HIV-1 and HIV-2 cores in TRIM5α expressing HeLa cells. *In situ* uncoating assay was performed in HeLa cells stably transfected with vector encoding HA-tagged cynomolgus monkey (CM) TRIM5α (TRIM5α; closed circles) or empty vector (Vc; opened circles). Cells were spinoculated with VSV-G-pseudotyped and GFP-Vpr/S15-dTomato-containing HIV-1 (NL-Nh/GFP-Vpr), or with VSV-G-pseudotyped GFP-Vpx/S15-dTomato-containing HIV-2 (GH123-Nh/GFP-Vpx or ASA-Nh/GFP-Vpx), and fixed at 0, 1, 2, or 4 hr after infection. CAs of the infected viruses were labeled by Cy5-conjugated viral-specific anti-CA antibodies, and the GFP punctae then were quantified and individually examined for the presence of dTomato (S15) and Cy-5 (CA) signals. The percentage of the number of productively entered (S15-negative) virions that stained for p24 or p26 CA among total number of productively entered virions (GFP-positive, S15-negative, and CA-positive particles/(GFP-positive, S15-negative, and CA-positive particles + GFP-positive, S15-negative, and CA-negative particles)) over time following fusion is shown in the Y-axis. The 0-hr time point and bafilomycin A-treated Vc-transfected cells (Vc+Baf; open squares) represent the total number of GFP-positive particles (both S15-positive and S15-negative) that stained positive for CA among total number of GFP-positive particles. The results shown are means and SDs of three independent cell lines at each time point. *, *p* value less than 0.02 when compared with values from Vc-transfected cells.

*Env*-deficient HIV-1 NL4-3, NL-Nh, was labeled with GFP-Vpr and S15-dTomato. Bafilomycin A (BafA), which blocks fusion of these viral and cellular membranes, was used in the negative control. This control was used to confirm that unfused viral particles failed to undergo uncoating. Infected cells were fixed and stained with the antibody against p24 CA. The data were graphed at each time point as a percentage of productively entered and CA-positive (coated) particles among total productively entered particles ((GB/ (GB+G) (%), Fig. 3).

In Vc-transfected cells, 54.4, 35.8, and 22.2% of productively entered HIV-1 particles were CA-positive at 1, 2, and 4 hr, respectively, after spinoculation (Fig. 3, left graph). This progression is in good agreement with our previous report, in which we had used unmodified wild-type HeLa cells [4]. In the presence of CM TRIM5α, however, HIV-1 infection yielded apparently smaller percentages of CA-positive particles, since 32.2, 23.7, and 13.1% of productively entered HIV-1 particles were CA-positive at 1, 2, and 4 hr, respectively, after spinoculation (Fig. 3, left graph). However, the difference was statistically significant only at 1 hr after spinoculation (*p* <0.005); differences fell short of significance at 2 and 4 hr (*p* = 0.15 and *p* = 0.053, respectively). On the other hand, at 0 hr (i.e., immediately after spinoculation), there was no difference in the percentage of CA-positive particles regardless of the presence or absence of CM TRIM5α. These results indicated that CM TRIM5α hastened the HIV-1 CA uncoating rate in HeLa cells. These observations are in good agreement with the results of previous studies, in which CA uncoating was biochemically analyzed in lysates of infected cells [15].

**Fig 4:**
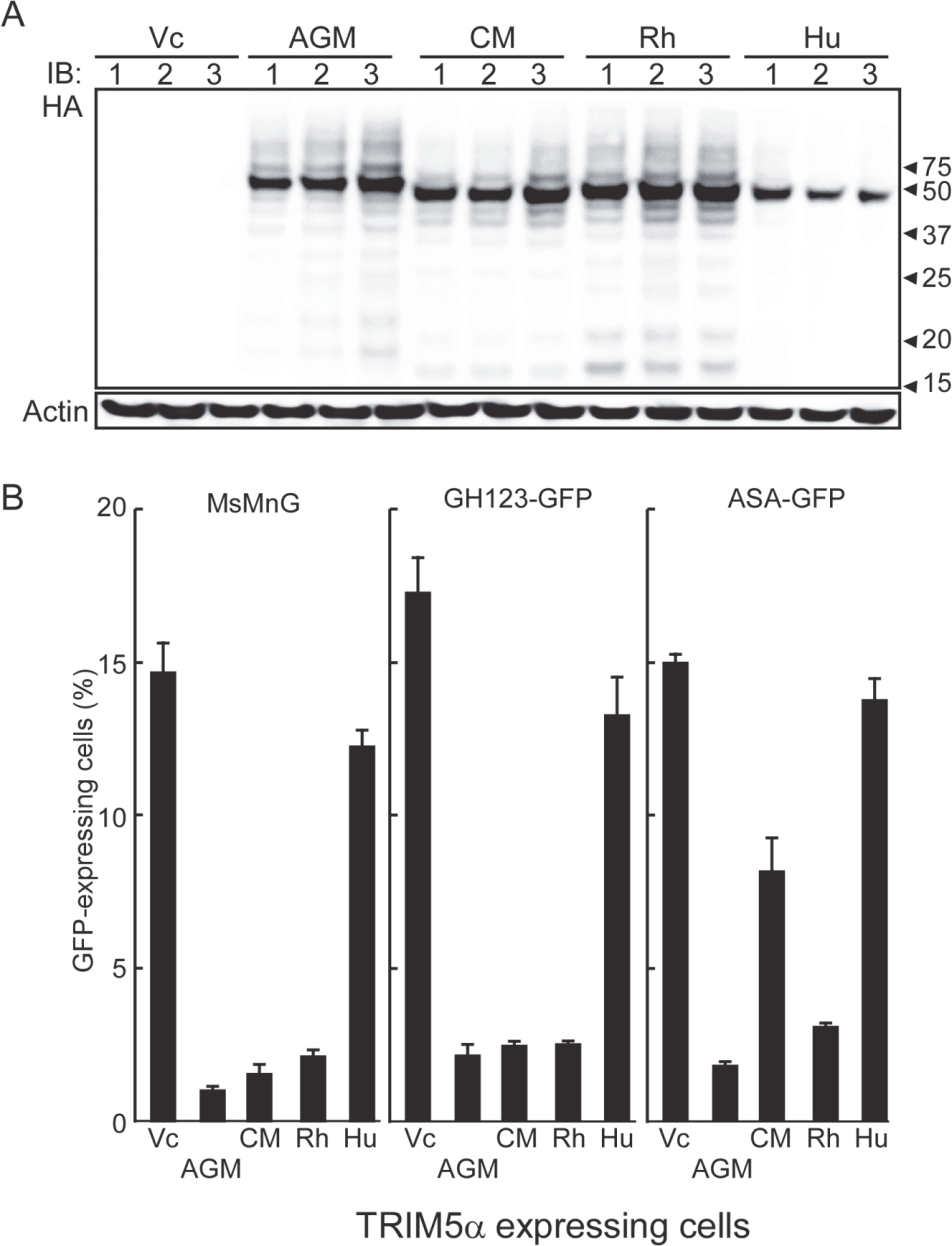
HeLa cell lines stably transfected with empty vector control (Vc) or with vector encoding HA-tagged TRIM5α homologs from African green monkey (AGM), rhesus macaque (Rh), cynomolgus monkey (CM), or human (Hu). (A) Three independent cell lines were tested for TRIM5α from each species (or empty vector, Vc). Lysates from each line were separated on a 4–20% gradient gel, transferred to a membrane, and probed with an anti-HA antibody (for HA-tagged proteins; upper panel) or anti-actin antibody (loading control; lower panel). (B) HeLa cells stably transfected with Vc or TRIM5α-encoding vectors were infected with GFP-encoding HIV-1 (MsMnG) or HIV-2 (GH123-GFP and ASA-GFP). GFP-positive cells were analyzed by flow cytometry as described above. Percentages of GFP-positive cells are shown as the means + SDs of the three independent cell lines.

We repeated our assay using HIV-2 strain GH123 and its variant ASA, which is resistant to human [19] and CM TRIM5α (see Fig. 4B). These HIV-2 particles were labeled with GFP-Vpx and S15-dTomato. CA-positive particles of GH123 constituted approximately 32% of productively fused virions at 1 hr after spinoculation in Vc-transfected cells, and the percentage of CA-positive particles did not appear to change with increased time, since 28.7 and 31.3% of productively fused virions were CA-positive at 2 and 4 hr after spinoculation (Fig. 3, middle graph). As with HIV-1, numbers of CA-positive particles decreased more rapidly in CM TRIM5α-expressing cells than in Vc-transfected cells. At 1, 2, and 4 hr after spinoculation, percentages of CA-positive particles were 16.6, 14.5, and 12.2%, respectively, in CM TRIM5α-expressing cells (Fig. 3, middle graph). Differences between presence and absence of CM TRIM5α were statistically significant at 1 and 4 hr after spinoculation (*p* = 0.014, and *p* = 0.008, respectively), but fell short of significance at 2 hr (*p* = 0.053). The sample images in Fig. 2 show the same trend, since four out of 13 particles were still coated in Vc-transfected cells (Fig. 2A), while only two out of 15 particles were coated in CM TRIM5α-expressing cells at 1 hr after spinoculation (Fig. 2B). The difference was evident even at 0.5 hr after spinoculation (S2 Fig., left graph). When we used CM TRIM5α-resistant HIV-2 variant ASA, percentages of CA-positive particles were 20.6% in Vc cells and 23.3% in CM TRIM5α-expressing cells at 1 hr after spinoculation, and there was no difference in percentages of CA-positive particles at 2 and 4 hr after spinoculation when comparing Vc cells to CM TRIM5α-expressing cells (Fig. 3, right graph). Similarly, the difference was not observed at 0.5 hr after spinoculation (S2 Fig., right graph). These results indicated that CM TRIM5α promoted accelerated uncoating of CM TRIM5α-sensitive HIV-2 strain GH123, but failed to affect the uncoating kinetics of CM TRIM5α-resistant HIV-2 variant ASA. These results are in good agreement with previous studies on HIV-1 [4], but represent the first demonstration that TRIM5α-sensitive HIV-2 uncoating also was accelerated in the presence of the respective TRIM5α.

In addition, we analyzed GFP-positive area sizes in all of the images we obtained and calculated the median and the interquartile range (IQR) of the area sizes (Table B in S1 File). The areas of GFP-positive particles at 0 hr were consistently larger than those at other time points in HIV-1-, HIV-2 GH123-, and HIV-2 ASA-infected cells (Table B in S1 File) The difference in area likely was due to the presence or absence of envelope around the core, because almost all the GFP-positive particles at 0 hr retained their envelope (S15-positive) just after adhesion to the host cell membrane, while more than 56% of GFP-positive particles had already lost their envelopes at 1 hr after spinoculation. Thus, this result suggests that GFP-Vpr and GFP-Vpx existed not only within the HIV core but also outside of the HIV core. When HIV invaded the host cell (by membrane fusion between the viral envelope and the cellular membrane), GFP-Vpr and GFP-Vpx outside of the core presumably were released into the cytoplasm before the core was uncoated. On the other hand, there was no difference in area sizes of GFP-positive particles between CA-positive and CA-negative particles (data not shown), suggesting that GFP-Vpr and GFP-Vpx form some sort of complex, presumably with other viral components, inside of the core.

### Restriction abilities of human and primate TRIM5αs against HIV-1 and HIV-2

To compare the effects on HIV-1 and HIV-2 uncoating of TRIM5α homologs from various primate species, we established new HeLa transfectants expressing AGM, CM, Rh, or Hu TRIM5αs as described above. Three independent cells lines were established for the TRIM5α derived from each species, and the expression of each TRIM5α protein was evaluated by immunoblotting (Fig. 4A). Each transfectant produced a TRIM5α protein of the expected size. However, compared with the OWM TRIM5αs, lower amounts of the Hu TRIM5α protein were detected in the corresponding cell lines. Therefore, we analyzed TRIM5α mRNA in these cell lines by semi-quantitative RT-PCR and found that all transfectants expressed TRIM5α-encoding mRNAs to similar levels (data not shown). These results are in a good agreement with a previous study showing that Hu TRIM5α accumulated to lower levels than Rh TRIM5α in transfected cells [24].

To determine the restriction activity against HIV-1 and HIV-2 in these established cell lines, we performed (as shown in Fig. 4B) a single-round infection assay similar to that of Fig. 1C. VSV-G-pseudotyped MsMnG, GH-123-GFP, or ASA-GFP viruses were inoculated into the Vc and TRIM5α-expressing transfectants; 24 hr after infection, cells were collected and fixed with formaldehyde, and GFP-expressing cell were counted by flow cytometry. In this experiment, approximately 15% of HeLa cells transfected with Vc were GFP-positive after infection with HIV-1, HIV-2 GH123, or HIV-2 ASA. Cells expressing AGM TRIM5α showed a strong restriction activity against all three viruses tested, with numbers of infected (GFP-positive) cells reduced to 1%, 2.2%, and 1.8% (respectively). Cells expressing CM or Rh TRIM5α homologs restricted HIV-1 and HIV-2 (GH123-GFP) similarly, with numbers of infected cells reduced to <2.5%. However, the CM-TRIM5α expressing cells were more permissive for ASA-GFP than were Rh TRIM5α expressing cells, with the CM cell line yielding 8.2% infected cells. On the other hand, cells expressing Hu TRIM5α showed the weakest restriction activity against HIV-1 and HIV-2 among TRIM5α-expressing lines, with the frequency of GFP-positive cells exceeding 12.3%. Notably, Hu TRIM5α-expressing cells showed no restriction activity against ASA-GFP. These results are in good agreement with our previous reports [18,19].

**Fig 5:**
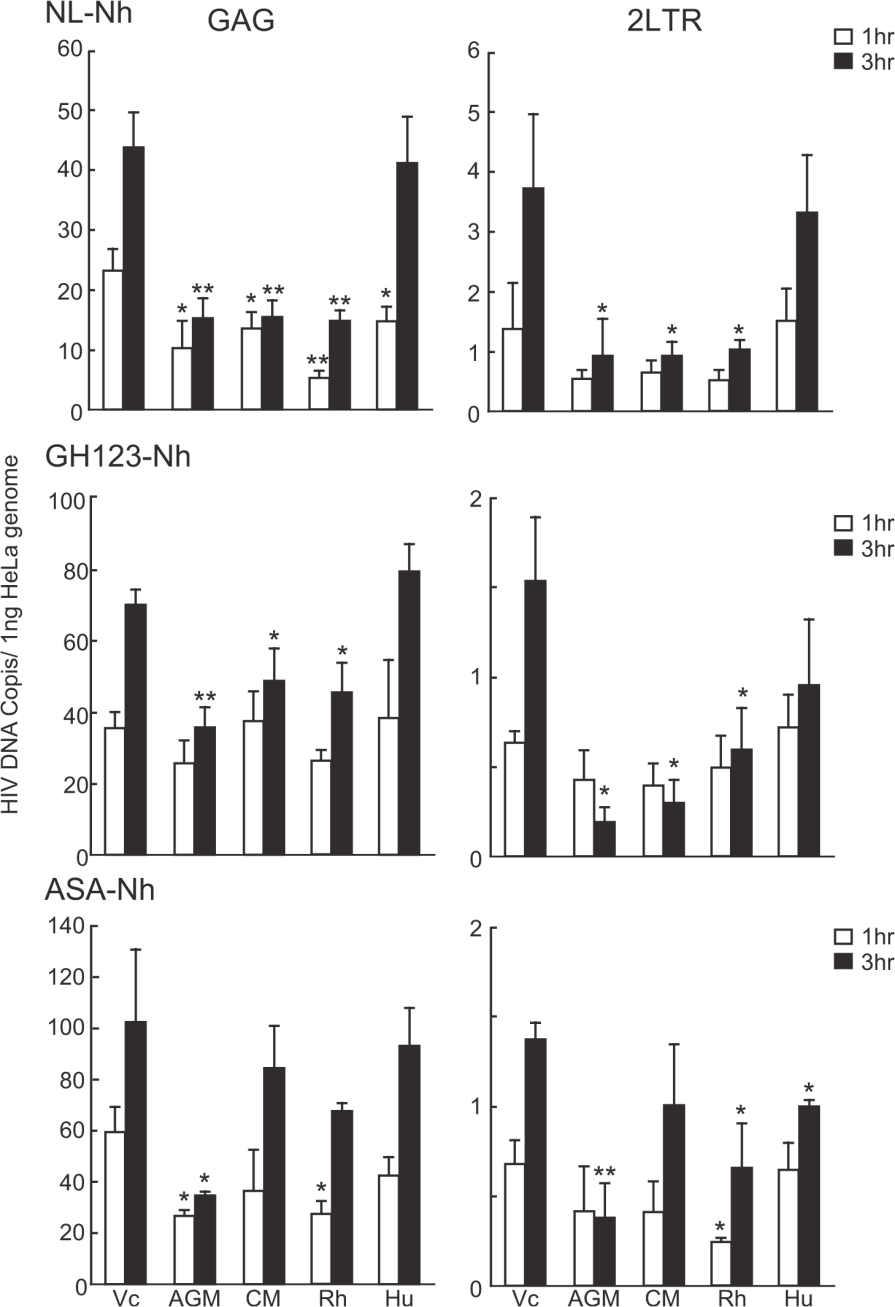
Measurement of the reverse-transcribed products of HIV-1 and HIV-2 in the TRIM5α-expressing HeLa cells. Whole DNA containing reverse-transcribed viral products was extracted from HeLa cell lines expressing HA-tagged African green monkey (AGM), rhesus macaque (Rh), cynomolgus monkey (CM), or human (Hu) TRIM5α, at 1 hr (white bars) and 3 hr (black bars) after spinoculation with VSV-G-pseudotyped HIV-1 (NL-Nh) or HIV-2 (GH123-Nh, ASA-Nh). HeLa cell lines stably transfected with empty vector (Vc) served as the positive controls in this assay. The extracted DNA was subjected to quantitative PCR using *GAG*-specific primers (for RT products) and 2-LTR-specific primers (for nuclear-transported viral DNA). Values were normalized to GAPDH-encoding DNA to yield viral DNA copy number per ng of total DNA. Results shown are the means + SDs of three independent cell lines. Note that viral DNA copy number was normalized per ng HeLa DNA (assessed as the copy number of the GAPDH-encoding gene in each sample); because human genomic DNA is present at approximately 1.0 to 2.0 pg per cell, one nanogram of HeLa DNA should correspond to the genomic DNA content of approximately 1,000 cells. * and **, *p* value less than 0.05 and 0.005, respectively, when compared with values in Vc-transfected cells at the respective time points.

We then measured RT production of VSV-G-pseudotyped NL-Nh, GH123-Nh, and ASA-Nh in HeLa transfectants expressing Hu or OWM TRIM5α. At 1 and 3 hr after spinoculation of HeLa cells, qPCR was used to quantify the late RT products (*GAG* DNA) and the formation of 2-LTR circles (a surrogate for nuclear transportation) (Fig. 5). Both late RT product synthesis and nuclear entry were decreased in TRIM5α-expressing HeLa cells. However, Hu TRIM5α-expressing HeLa cells showed weaker restriction compared to cells expressing OWM TRIM5αs. In the case of VSV-G pseudotyped ASA-Nh, there was no significant difference in late RT and 2-LTR product levels among HeLa cells expressing CM or Hu TRIM5α and those transfected with Vc. These results confirmed and extended the previous notion that OWM TRIM5αs restrict TRIM5α-sensitive lentiviruses at an early stage of infection.

### Uncoating rate of HIV-1 and HIV-2 in OWM and Hu TRIM5α-expressing HeLa cells

**Fig 6:**
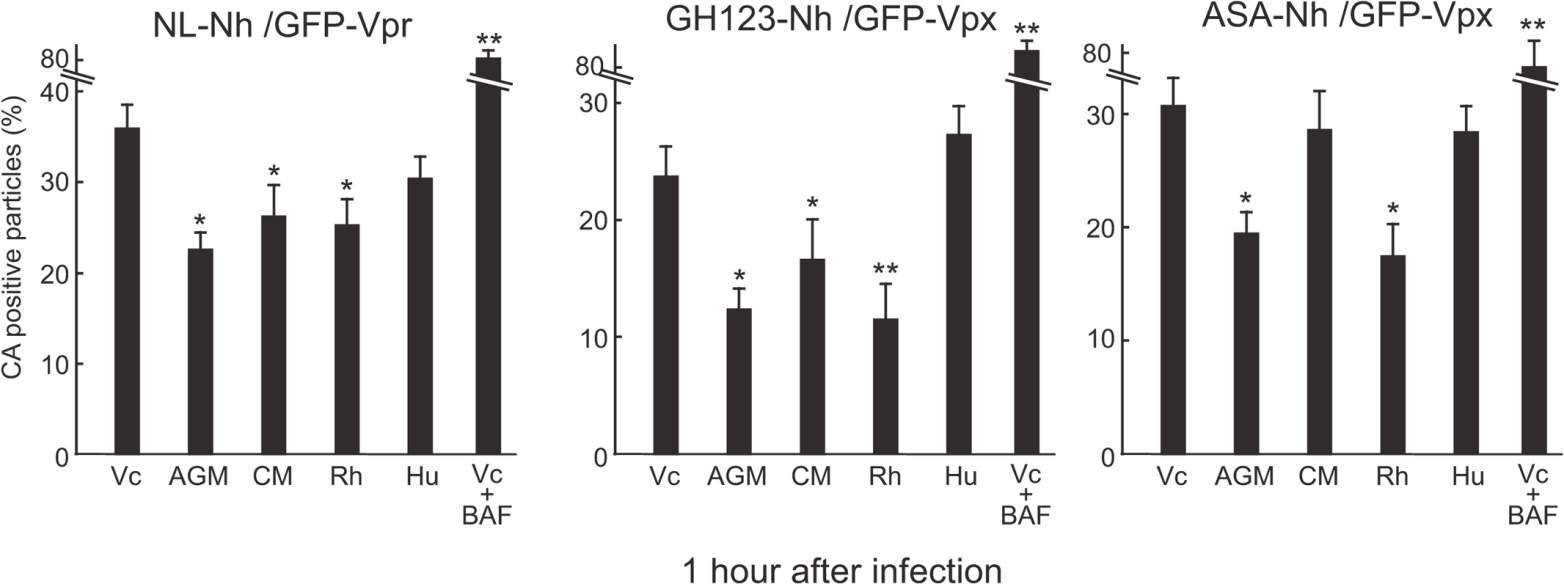
Uncoating rates of HIV-1 and HIV-2 cores in HeLa cell lines stably transfected with empty vector control (Vc) or with vector encoding HA-tagged TRIM5α from African green monkey (AGM), rhesus macaque (Rh), cynomolgus monkey (CM), or human (Hu). *In situ* uncoating assay was performed in multiple HeLa cell lines derived from each construct. At 1 hr after spinoculation with fluorescently labeled HIV-1 (NL-Nh/GFP-Vpr) or HIV-2 (GH123-Nh/GFP-Vpx or ASA-Nh/GFP-Vpx), cells were fixed. The infecting viruses and GFP punctae were visualized and analyzed as described in the legend for Fig. 3. Data shown in the Y-axis are percentage of the number of productively entered (S15-negative) virions that stained for p24 or p26 CA among total number of productively entered virions (GFP-positive, S15-negative, and CA-positive particles/(GFP-positive, S15-negative, and CA-positive particles + GFP-positive, S15-negative, and CA-negative particles)). The bafilomycin A-treated Vc cells (Vc+BAF) were used as a negative control and represent percentage of the total number of GFP-positive particles (both S15-positive and S15-negative) that stained positive for CA among total number of GFP-positive particles. The results shown are means + SDs of three independent transfected cell lines. * and **, *p* value less than 0.05 and 0.005, respectively, when compared with values in Vc-transfected cells.

To examine the uncoating rate of HIV-1 and HIV-2 in the presence of TRIM5α from different primate species, we measured percentages of CA-positive GFP particles at 1 hr after spinoculation in CM, Rh, AGM, or Hu TRIM5α-expressing HeLa cells. Percentages of CA-positive GFP particles in cells that had been transfected with Vc-in the presence of BafA served as a negative control for this uncoating assay (Fig. 6). In this experiment, the frequency of CA-positive GFP particles in GFP particles lacking S15 was 36% (NL-Nh), 24% (GH-123-Nh), or 31% (ASA-Nh) upon infection of Vc-transfected cells. In AGM and Rh TRIM5α-expressing cells, the corresponding frequencies were significantly decreased, with values falling to 22.3% (*p* = 0.011) with NL-Nh, 12.3% (*p* = 0.036) with GH123-Nh, and 19.5% (*p* = 0.008) with ASA-Nh upon infection of AGM TRIM5α-expressing cells, and to 25.3% (*p* = 0.012), 11.6% (*p* = 0.004), and 17.4% (*p* = 0.010) (respectively) upon infection of Rh TRIM5α-expressing cells (P values represent comparison, for a given virus, of Vc cells to those expressing TRIM5α). In CM TRIM5α-expressing cells, the frequencies of CA-positive GFP particles were significantly decreased in NL-Nh- (26.3%, *p* = 0.022) and GH123-Nh-infected cells (16.7%, *p* = 0.023), but not in ASA-Nh-infected cells (28.7%, *p* = 0.547), confirming a distinction observed above in Figs. 3 and 4B. On the other hand, in Hu TRIM5α-expressing cells, frequencies of CA-positive GFP particles were not significantly decreased (30.5% in NL-Nh-, 27.3% in GH123-Nh-, and 28.5% in ASA-Nh-infected cells) in comparison with those in Vc expressed cells. These results indicated that TRIM5α-sensitive lentivirus showed accelerated uncoating in the presence of certain TRIM5αs, while uncoating of TRIM5α-resistant lentiviruses was not affected by the respective TRIM5α.

## Discussion

In the present study, we report the adaptation of an *in situ* uncoating assay to HIV-2 by modification of a method previously reported for HIV-1 [4,5]. We used a SIVsm-derived GFP-Vpx fusion and a monoclonal antibody against HIV-2 CA in place of (respectively) an HIV-1-derived GFP-Vpr fusion and a monoclonal antibody against HIV-1 CA. We also showed that both HIV-1 NL4-3 and HIV-2 GH123 exhibited accelerated uncoating in the presence of OWM TRIM5s. Furthermore, a CM TRIM5α-resistant HIV-2 variant, GH123 ASA, did not show accelerated uncoating in the presence of CM TRIM5α, confirming that accelerated uncoating is associated with restriction activity of TRIM5α against HIV.

In the presence of OWM TRIM5αs, accelerated uncoating was evident at 1 hr after spinoculation (Figs. 3 and 6) and continued to be evident at 4 hr after spinoculation (Fig. 2). The kinetics of accelerated uncoating in the present study was in good agreement with that in previous reports [4,5]. In those reports, uncoating speed was assayed by measuring the amount of CA protein, which possesses a density equivalent to that of the assembled capsid, after sucrose density gradient centrifugation of infected cell lysates. The density method, however, cannot differentiate productively entered HIV particles from those that were simply endocytosed, since whole cells were lysed and directly subjected to sucrose density gradient centrifugation. Therefore, the *in situ* uncoating assay described here reveals, for the first time, that OWM TRIM5αs cause accelerated uncoating of productively entered HIV-1 and HIV-2 particles.

In the present study, we also established a semi-automatic counting system for use in the *in situ* uncoating assay. This system not only allowed us to count particles in a convenient and speedy way, but also ensured that we were counting particles in a consistent and unbiased fashion in all of the obtained images. Comparison of results obtained by the semi-automated counting system with those obtained by the previous manual counting method showed a high correlation coefficient, confirming the equivalence of these two methods (Table A in S1 File). The semi-automatic counting system also allowed us to compare the sizes of fluorescent particles among images obtained in a given experiment. As shown in Table B in S1 File, GFP-labeled particles of both HIV-1 and HIV-2 apparently became smaller after productive entry into the host cells. Both HIV-1 Vpr and HIV-2/SIV Vpx have been shown to bind to the p6 domain of the Pr55^gag^ protein and are incorporated into virus particles [25–27]. Vpr in viral particles is necessary for the efficient translocation of the PIC into the nucleus and subsequent infection in non-dividing cells [28–30]. HIV-1 Vpr and HIV-2 Vpx have been found in the mature cores of the respective viruses [26,31]. Therefore, it is tempting to speculate that HIV-1 Vpr and HIV-2 Vpx are packaged not only inside of, but also outside of, the virion cores; only Vpr and Vpx packaged outside of the core would be released into the cytoplasm when HIV productively enters into host cells. Further experiments will be needed to test this hypothesis.

When we compared uncoating kinetics between HIV-1 NL4-3 and HIV-2 GH123 in the absence of OWM TRIM5α, HIV-2 GH123 tended to show faster uncoating kinetics than did HIV-1 NL4-3: at 1 hr after spinoculation, more than 50% of HIV-1 NL4-3 particles that had productively entered cells remained coated, while less than 35% of HIV-2 GH123 or GH123 ASA particles that had productively entered cells were coated (Fig. 3). This effect also was evident in the independent experiments shown in Fig. 6. At present, the basis for the difference in uncoating kinetics between HIV-1 NL4-3 and HIV-2 GH123 is unclear. A difference in anti-CA antibody affinity may affect the uncoating kinetics, since we used distinct monoclonal antibodies against HIV-1 and HIV-2 CA proteins. However, we do not favor this explanation, given that the percentages of coated HIV-2 GH123 particles remained stable after 1 hr, while those of HIV-1 NL4-3 continued to decrease until 4 hr. Alternatively, differences in the GFP-labeled virus accessory proteins (Vpr and Vpx) may affect the uncoating kinetics, although we note that there was no difference in the sizes of GFP-positive particles between HIV-1 NL4-3 and HIV-2 GH123. Therefore, we presume that the uncoating kinetics of HIV-2 GH123 are indeed faster than that of HIV-1 NL4-3. Further studies, including *in situ* uncoating assays using other HIV-1 and HIV-2 strains, are needed before we can generalize from this observation.

Fig. 4A shows that levels of human TRIM5α protein expression in established transfectants were lower than those of OWM TRIM5αs. This result is in good agreement with our previous report, in which human TRIM5α was expressed using an SeV vector [32]. However, levels of the human TRIM5α-encoding RNA were similar to those of other TRIM5α-encoding transcripts when assayed by semi-quantitative RT-PCR. Human TRIM5α- and other TRIM5α-encoding genes were expressed under control of the same CMV promoter in the pCEP4-vector. These results and those of the previous study [24] suggested that human TRIM5α protein exhibits lower stability than OWM TRIM5αs in human cells. The question of whether OWM TRIM5αs also exhibit prolonged stability in their respective OWM cells will require further investigation.

Very recently, it was reported that RT reactions may be dispensable for uncoating and nuclear import of the PIC [33]. Authors of that communication used an RT-deficient HIV-1 isolate that contained fluorescently labeled APOBEC3F. However, when those authors used RT-intact wild-type HIV-1, only ∼6% of fluorescent signals in the nuclei co-localized with HIV-1 RNA signals [33]. These results, together with those of the previous study [6], indicate that HIV core plays an important role in RT and the establishment of infection. These issues will need to be clarified in future studies.

In conclusion, we adapted an HIV-1 *in situ* uncoating assay to HIV-2 and showed that both HIV-1 and HIV-2 exhibited accelerated uncoating in the presence of OWM TRIM5αs. We anticipate that the use of this imaging technology in our virus uncoating assay will permit us to use dyes of distinct emission wavelengths to distinguish the roles of various host factors involved in uncoating.

## Supporting Information

S1 Fig HIV-2 uncoating images at lower magnification. Fluorescently labeled HIV-2 were spinoculated into empty vector-transfected (Vc/HeLa) or cynomolgus monkey TRIM5α-expressing (CM TRIM5α/HeLa) HeLa cells. Cells then were treated and analyzed as described in the legend for Fig. 2. Merged images of GFP-Vpx, S15-dTomato, and HIV-2 P26 fluorescence are shown. Bars indicate 20 μm. Dashed lines indicate the regions shown at higher magnification in Fig. 2.
(EPS)

S2 Fig Uncoating kinetics of HIV-2 cores in TRIM5α-expressing HeLa cells in a short time-course. *In situ* uncoating assay was performed in HeLa cells stably transfected with vector encoding HA-tagged cynomolgus monkey (CM) TRIM5α (TRIM5α; closed circles) or empty vector (Vc; opened circles). Cells were spinoculated with VSV-G-pseudotyped GFP-Vpx/S15-dTomato-containing HIV-2 (GH123-Nh/GFP-Vpx or ASA-Nh/GFP-Vpx), and fixed at 0, 0.5, or 1 hr after infection. HIV-2 CAs were labeled after fixing the cells, and then analyzed as described in the legend for Fig. 3. The results shown are means ± SDs of three independent cell lines at each time point. *, *p* value less than 0.05 when compared with values from Vc-transfected cells.
(EPS)

S1 File Table A, Comparison between semi-automatic and manual counting. Table B, Median GFP areas.
(DOCX)
