# Supplementary material for: Fluorescent Image Analysis of HIV-1 and HIV-2 Uncoating Kinetics in the Presence of Old World Monkey TRIM5α
Source: PLoS One. 2015 Mar 24;10(3):e0121199. doi: 10.1371/journal.pone.0121199 (PMC4372348; doi:10.1371/journal.pone.0121199)
Supplement: S1 File — Table B, Median GFP areas. (DOCX) [file pone.0121199.s003.docx]

Supporting Table A. Comparison between semi-automatic and manual counting

|  | Total particles (RGB+GB+G) | | Productively entered particles (GB+G) | | Coated particles (RGB for 0hr and GB for 1-2hr) | | (RGB+GB) /(RGB+GB+G) (%) | | GB/(GB+G) (%) | | Missing count of total particles (%) |
| --- | --- | --- | --- | --- | --- | --- | --- | --- | --- | --- | --- |
| Methods | Semi-auto | Manual | Semi-auto | Manual | Semi-auto | Manual | Semi-auto | Manual | Semi-auto | Manual | (Manual-Semi-auto)/Manual |
| 0 hr | 196 | 232 |  | | 160 | 190 | 81.6 | 81.9 |  | | 15.5 |
|  | 115 | 157 |  |  | 96 | 135 | 83.5 | 86.0 |  |  | 26.8 |
|  | 166 | 200 |  |  | 124 | 152 | 74.7 | 76.0 |  |  | 17.0 |
| 1-2 hr | 239 | 248 | 164 | 189 | 87 | 93 |  | | 53.0 | 49.2 | 3.6 |
|  | 104 | 145 | 89 | 113 | 56 | 68 |  |  | 62.9 | 60.2 | 28.3 |
|  | 197 | 212 | 135 | 176 | 92 | 123 |  |  | 68.1 | 69.9 | 7.1 |
|  | 97 | 112 | 84 | 108 | 33 | 46 |  |  | 39.3 | 42.6 | 13.4 |
|  | 60 | 82 | 48 | 72 | 21 | 33 |  |  | 43.8 | 45.8 | 26.8 |
|  | 92 | 118 | 89 | 112 | 42 | 49 |  |  | 47.2 | 43.8 | 22.0 |
| r^2^ | 0.9797 | | 0.9903 | | 0.9861 | | 0.9761 | | 0.9589 | |  |
| Mean±SD |  | | | | | | | | | | 17.8±8.37 |

RGB: red, green, and blue particles

GB: green and blue particles

B: blue particles

r^2^: correlation coefficient

SD: standard deviation

Supporting Table B. Median GFP areas

|  |  | Empty vector (Vc) | | | | Cynomolgus monkey TRIM5α | | | |
| --- | --- | --- | --- | --- | --- | --- | --- | --- | --- |
|  |  | GFP  number | median | IQR | p value | GFP  number | median | IQR | p value |
| NL-Nh | 0H | 1757 | 0.28 | (0.14-0.53) |  | 2520 | 0.42 | (0.22-0.70) |  |
|  | 1H | 1743 | 0.20 | (0.09-0.37) | ** | 1039 | 0.20 | (0.10-0.38) | ** |
|  | 2H | 627 | 0.17 | (0.09-0.35) | ** | 702 | 0.15 | (0.08-0.31) | ** |
|  | 4H | 361 | 0.17 | (0.08-0.39) | ** | 781 | 0.17 | (0.09-0.34) | ** |
| GH123-Nh | 0H | 2060 | 0.23 | (0.13-0.44) |  | 1801 | 0.24 | (0.13-0.45) |  |
|  | 1H | 813 | 0.20 | (0.10-0.51) |  | 786 | 0.16 | (0.07-0.45) | ** |
|  | 2H | 1227 | 0.18 | (0.08-0.43) | ** | 825 | 0.18 | (0.09-0.43) | * |
|  | 4H | 781 | 0.17 | (0.09-0.40) | ** | 656 | 0.18 | (0.09-0.40) | ** |
| ASA-Nh | 0H | 3110 | 0.17 | (0.08-0.37) |  | 2231 | 0.23 | (0.12-0.45) |  |
|  | 1H | 1525 | 0.15 | (0.08-0.35) |  | 944 | 0.16 | (0.08-0.45) | ** |
|  | 2H | 1041 | 0.14 | (0.07-0.34) | * | 622 | 0.21 | (0.09-0.49) |  |
|  | 4H | 754 | 0.12 | (0.07-0.29) | ** | 944 | 0.18 | (0.08-0.43) | ** |

Numbers listed are GFP-particle totals in three independent cell lines. Median and IQR (interquartile range) of GFP-particle areas (μm^2^) are shown. * p < 0.05 and ** *p* < 10^-5^ when compared with those at 0 hr (Mann-Whitney U test with Bonferroni correction).
